# Editorial

**Published:** 1865-11

**Authors:** 


### Editorial.

---

### DUODENO-HEPATITIS, OR ACUTE JAUNDICE. THE SUBSTANCE OF A CLINICAL LECTURE IN THE MERCY HOSPITAL, OCTOBER 23D, 1865.

BY N. S. DAVIS, M.D., Prof. Clin. Med., &c., Chicago, Ill.

---

GENTLEMEN:—The patient before you is a man, aged about 26 years, who was admitted to the hospital three days since. He had been sick two weeks previously. You see, at a glance, that his whole cutaneous surface is of a deep yellow color; the conjunctiva is as yellow as though painted with gamboge. The skin over the epigastrium is not only deeply yellow, but presents numerots small red spots, like minute spots of extravasated blood. The extremities are cool; the expression of countenance dull; lips dry; tongue moderately coated with a brownish fur; pulse small, weak, and 110 per minute; stomach so irritable as to reject, by vomiting, most of the nourishment or drink given to the patient; bowels quiet, but not constipated; urine scanty and of a very dark, reddish-brown color. Patient complains of great weakness and weariness, with a sense of oppression at the epigastrium. Though the universal distribution of the coloring matter of bile, so visible in the whole exterior of the patient, indicates some disease or obstruction connected with the liver, yet you perceive that a close physical exploration, by palpation and percussion, affords no evidence of enlargement of that organ; but we find decided tenderness to percussion or firm pressure over that part of the epigastrium corresponding with the position of the doudenum and hepatic ducts.

What is the nature of the disease under which this patient is suffering? Many of you will be prompt to answer, in popular language, that it is a plain case of jaundice. But jaundice simply means yellow, and is, like dropsy, a mere symptom. The term gives us no information in relation to the pathology of the case. The jaundice, or yellow color, is merely a symptom, indicating the retention of the coloring matter of bile, and its deposit in the various structures of the body. It does not, with certainty, indicate any form of hepatic disease; much less that the secretory function of the liver is torpid or inactive. On the contrary, the abundant diffusion of bile throughout the system, not only coloring the tissues, but passing off freely in the urine, perspiration, &c., shows that the secreting cells of the liver so far perform their office as to cause the elements of bile to unite and form an abundance of that fluid, but it does not pass off through its natural channel, the hepatic duct. Hence, while the conjunctiva, skin, urine, &c., are all highly colored, the fœces are clay-colored, or whiter than natural. This failure of the bile to pass off through the hepatic ducts may arise from three widely different pathological conditions. It may be caused by such an alteration in the sensibility or properties of the capillary extremities of the hepatic ducts as will prevent them from taking up the bile as it is secreted in the lobules of the liver, leaving that office to be performed by the venous capillaries through which the bile passes back into the blood, instead of through the ducts to the duodenum. The second condition is that of simple mechanical obstruction of the larger hepatic ducts. The most common cause of this, is the formation of biliary calculi. The obstruction from this cause may be only temporary, as when a calculus is delayed in its passage, creating great pain, sympathetic gastric irritation, and general depression, ending suddenly when the calculus finally passes from the duct into the duodenum. Or it may be permanent, as when similar calculi accumulate in the gall-bladder and remain there for years. But the third, and most common cause of jaundice, is a subacute inflammation of the mucous membrane lining the duodenum and the *ductus communis coledochus.*

You are aware that the two constant or essential elements of inflammation are, morbid excitability of the diseased structure, and accumulation of blood in its capillaries; and the most constant accompanying effect is increased bulk or tumefaction. If you remember that the *ductus communis* is scarcely larger than a crow's quill, and that it opens into the intestines obliquely, you will readily perceive that a very moderate tumefaction of the mucous membrane, lining that part of the duodenum where the duct opens, would be sufficient to obstruct the opening and render the passage of bile very slow, or stop it altogether. And if the inflammation causing the tumefaction should extend to the lining membrane of the duct also, you readily perceive how it would almost necessarily render the obstruction complete while such inflammation should continue. It is to this last class of cases that the patient before you belongs. It is, indeed, a very strongly marked representative of the class.

Let us examine a little more closely the essential phenomena or symptoms of this case. If you note the sounds carefully, as I percuss, you will readily detect the line where pulmonary resonance ends and hepatic dulness begins, and, as I move downwards, you will distinguish, still more plainly, where the latter dulness ends and the intestinal resonance begins. You are thus enabled to measure the exact vertical depth of the liver. If we extend the percussion to the left, over the epigastric region, we again find the hepatic dulness ceasing before we reach the centre of the latter region, thus determining the limits of the left lobe. By this examination, you learn that the liver, in this case, occupies no more than its natural space. And you also learn another thing of great importance, namely, the exact location and extent of tenderness. While percussion was going on over the right hypochondriac region, occupied by the liver, you perceived no indications of pain or soreness manifested by the patient; but so soon as the act extended to the right margin of the epigastric region, the patient began to give signs of suffering. In this patient there is decided tenderness over the whole epigastric region, but more acute at the right lower margin of that region, which is directly over the point where the biliary duct enters the duodenum. You will find some tenderness in that locality in almost every case of this variety of disease. There is also a sense of heaviness, or oppression, that is annoying to the patient, and is generally increased, with some feeling of nausea, from half to three-quarters of an hour after taking food. In the patient before you, this feeling of oppression and disposition to vomit is more strongly manifested than is usual in the class of cases to which it belongs. All the important symptoms point to the duodenum and common hepatic duct, as the principal seat of the disease; and they equally point to a mild grade of inflammation, as the form of disease existing. The disturbed condition of the digestion, especially the latter stages of it, with frequent nausea, sense of heaviness, and tenderness in the right and lower part of the epigastrium, clearly indicate both the seat and the inflammatory nature of the disease. As this inflammation is usually of a mild grade, it seldom causes the death of the patient, and consequently the opportunities for *post mortem* examinations have been few.

The disease occurs most frequently in the Autumn, during the first cold and wet weather. In this city, we meet with cases every year, during the months of October and November; and sometimes the cases are so numerous as to well-nigh merit the title of epidemic. About ten years since, during the Autumn, the disease was so prevalent in this city, that more than sixty cases came under my care within thirty days. A few of the cases in that season assumed an unusual degree of severity. The only fatal case that occurred directly in my own practice, was that of a female six months advanced in pregnancy. The disease commenced with the usual symptoms, and after the system had become thoroughly impregnated with bile, the secretions and whole cutaneous surface deeply yellow, the gastric irritability became so great that nothing was retained on the stomach, the urinary secretion diminished, day by day, until it was entirely suppressed; the surface assumed a darker, more bronzed hue; the mental dulness increased, until it became coma; and in that state uterine contractions commenced, expelling the fœtus and placenta, followed by the loss of only a few ounces of blood, and without the slightest apparent consciousness of the patient. A few hours after this, the acts of vomiting became more like an involuntary regurgitation of a large quantity of a dark coffee-ground fluid, closely resembling the black-vomit of yellow fever. The patient died in a few hours. No *post mortem* examination was allowed in that case; but during the same season, a fatal case occurred in the practice of a neighboring physician, which was uncomplicated with pregnancy, and in which a *post mortem* examination was allowed. No important local lesions were found, except in the mucous membrane of the duodenum, hepatic ducts, and central portion of the liver. The mucous membrane of the duodenum was intensely red throughout its whole extent, except some spots where it had assumed a dark hue. It was thickened or tumefied, and, in the darker colored places, softened. The membrane lining the hepatie ducts from the orifice in the duodenum to the smaller branches in the liver, were in the same pathological condition. The gall-bladder was moderately distended with healthy-looking bile, and its interior surface appeared to be free from inflammation. No biliary calculi were found. The liver appeared to be very slightly enlarged, color natural over the convex surface, but paler than natural over a part of the under or concave surface, especially around the entrance of the vessels and ducts. This alteration of color extended into a portion of the interior structure, which seemed to be softened and moderately infiltrated with fat globules.

You will see that the results of this examination fully confirm the opinion, already expressed, as to the seat and inflammatory nature of the disease under which our present patient is laboring. If the patient is thus laboring under an inflammation of the mucous membrane of the duodenum and hepatic ducts, causing sufficient tumefaction to obstruct the free flow of bile, thereby inducing the jaundiced color; the oppression and tenderness in the epigastrium; the imperfect digestion, with frequent sense of nausea, and sometimes vomiting, what are the rational indications for treatment?

The patient feeling oppressed and sick in the epigastric region, and his friends seeing his eyes begin to look yellow, very naturally concludes that he is bilious, which term is used to express some vague idea of the retention of bile on the stomach, and, consequently, hastens to swallow a dose of active physic, or an emetic, or both, for the purpose of carrying it off. And we have occasionally met with physicians who had prescribed the same remedial agents, and followed their operation by other remedies directly calculated to act on the liver, to increase the secretion of bile.

After what has been said of the nature of this disease, and the fact that the bile does not exist in the stomach in any quantity, I need not say that such harsh evacuants as emetics and cathartics, especially in the early stage, are worse than useless. They not only fail to afford any of the relief that the patient expected, but they frequently so increase the irritation of the mucous membranes as to cause a persistent disposition to vomit, and sometimes diarrhœa. And to give remedies directly calculated to increase the secretion of bile before the obstruction had been removed from the ducts, would be as absurd as to give diuretics while the bladder was retained full of urine, by a stricture of the urethra.

The plain indications in this, and all similar cases, is to allay the irritation of the mucous membrane and diminish its vascularity, thereby removing the tumefaction, and, consequently, the obstruction of the ducts. It is desirable also to promote, as far as possible, the action of the skin and kidneys, that the excrementitious matters of the system may be more fully eliminated. These objects can usually be accomplished by very simple remedial measures. I usually commence the treatment of such cases by giving one of the following powders every four hours, until six have been taken:—

℞. Hydrarg. Chlorid. Mite, ------------- 10 grs.
Pulv. Doveri, ------------------------ 30 grs.
Nit. Potassa, ------------------------ 30 grs.

Mix, divide into six powders.

When these are all taken, if the bowels do not move spontaneously, a mild laxative should be given, after which the Dover's powder and nitrate of potassa may be resumed, and continued, with an occasional mild laxative, until the recovery is completed. In the milder class of cases, this will be all the treatment required. But if, as in this case, the stomach is so irritable as to induce frequent vomiting, the Dover's powder and nitrate potassa may be replaced by sulphate of morphia, one-quarter of a grain, and bi-carbonate of soda, 5 grains, in each powder, with leeches or counter-irritation over the epigastrium. If the urine has become very scanty, a teaspoonful of nitrous ether may be given between the powders. The nourishment should be bland and simple throughout the treatment; and, after the intestinal evacuations have become dark, green, or yellow, showing that the bile is passing freely through the ducts, if there is much debility, with impaired appetite, as is often the case, the mineral acids, especially the nitro-muriatic, may be given with much advantage. Sometimes there will be left a constant tendency to constipation. If so, one of the following pills may be given every night, or every night and morning, with benefit:—

℞. Ext. Hyoscyamus, ---------------------- 30 grs.
Ext. Taraxacum, ------------------------ 30 grs.
Sulph. Ferri, ----------------------------- 30 grs.
Pulv. Aloes, ------------------------------ 15 grs.
Ext. Nux Vom., ------------------------- 15 grs.

Mix, and divide into 30 pills.

Such is the course of treatment which has been commenced, and will be carried out in the case before you, the progress and results of which will be observed by you at subsequent clinics.

### PRACTICAL PHARMACY.

At the late meeting of the American Pharmaceutical Association, held in Boston, Dr. SQUIBB read a very interesting paper on Fluid Extracts which, if adopted by the Committee on Revision, will introduce a radical change in the above class of preparations. In the last edition of the Pharmacopea, the following plan was adopted as a basis for making nearly all the fluid extracts:—Sixteen ounces of a drug is directed to be percolated with from three to five pints of dilute alcohol, reserving and setting aside the first twelve ounces which pass through, the remainder is to be evaporated down to four fluid ounces, and the two solutions mixed together, making one pint of fluid extract. This certainly is a very excellent process, but unfortunately the high duty placed on alcohol rendered it impossible for the retail druggists to make these fluid extracts without sustaing a heavy loss, and in consequence, this branch of practical pharmacy fell into the hands of a few who manufactured them on a large scale, and were able to use suitable apparatus for collecting the alcohol again.

The following is a synopsis of Dr. SQUIBB's paper, taken from the *Chemist's & Druggist's Circular*, for October:—Sixteen ounces of a drug, as for instance colchicum, is to be percolated with alcohol of suitable strength, adding one pint, and as soon as the alcohol disappears from the top, water is poured on till fourteen fluid ounces of percolate is obtained, this constitutes the fluid extract, there is to be no evaporation and no excess of alcohol added, and the fluid extract is a strong and a great deal cheaper than if a gallon had been passed through it and evaporated down to a pint. Dr. SQUIBB also finds that fluid extracts, prepared either by this process or by that of the Pharmacopœia, are relatively stronger than the drugs from which prepared, while they are perhaps twice as strong as the majority of those sold by the leading manufacturers. The advantages of the above process, are:—First, it will entirely do away with both fluid extracts and the ordinary tinctures, replacing them with an article which can be made by any druggist, and will really represent a pint for a pound. Again, in consequence of no heat being used in the process, all the essential and volatile principles are retained unchanged; they can also be made for one-half the price of the present fluid extracts. These are very considerable advantages, and will certainly compensate for any confusions caused in adopting this new process.

Anticipating the decision of the Pharmaceutical Committee. I shall keep on hand a complete assortment of these concented tinctures for dispensing.

S. W. GILLESPIE,
Laboratory, Cor. Monroe and State Streets.

Chicago, Oct. 28, 1865.

